# Nutzung von Routinedaten aus Notaufnahmen zur Surveillance von Suizidversuchen und psychiatrischen Notfällen

**DOI:** 10.1007/s00103-021-03467-x

**Published:** 2021-12-10

**Authors:** Carmen Schlump, Julia Thom, T. Sonia Boender, Birte Wagner, Michaela Diercke, Theresa Kocher, Alexander Ullrich, Linus Grabenhenrich, Felix Greiner, Rebecca Zöllner, Elvira Mauz, Madlen Schranz

**Affiliations:** 1 Abteilung für Epidemiologie und Gesundheitsmonitoring, Robert Koch-Institut, Berlin, Deutschland; 2 Abteilung für Infektionsepidemiologie, Robert Koch-Institut, Berlin, Deutschland; 3 Methodenentwicklung und Forschungsinfrastruktur, Robert Koch-Institut, Berlin, Deutschland; 4 Universitätsklinik für Unfallchirurgie, Otto-von-Guericke-Universität Magdeburg, Magdeburg, Deutschland; 5 AKTIN-Notaufnahmeregister, Magdeburg/Aachen, Deutschland; 6 Gesundheitsamt Frankfurt am Main, Frankfurt, Deutschland

**Keywords:** Public-Health-Surveillance, Sekundärdaten, Mental-Health-Surveillance, Suizidalität, Notfallversorgung, Public health surveillance, Secondary data, Mental health surveillance, Suicidal tendencies, Emergency care

## Abstract

**Hintergrund:**

Die Häufigkeit von Suizidversuchen ist ein zentraler Indikator der psychischen Gesundheit der Bevölkerung und daher Gegenstand der Mental Health Surveillance am Robert Koch-Institut. Da bisher keine Datenquellen systematisch zur kontinuierlichen Erfassung von psychiatrischen Notfällen – zu denen Suizidversuche zählen – herangezogen werden, wird die Nutzung von Routinedaten aus Notaufnahmen zu diesem Zweck geprüft.

**Methoden:**

Routinedaten aus 12 Notaufnahmen wurden für den Zeitraum 01.01.2018–28.03.2021 ausgewertet. Syndromdefinitionen für *Suizidversuche, psychiatrische Notfälle *und* psychische Symptomatik *wurden als Kombinationen aus Vorstellungsgründen und Diagnosen entwickelt. Fälle wurden alters- und geschlechtsspezifisch sowie im Zeitverlauf dargestellt.

**Ergebnisse:**

Von insgesamt 1.516.883 Notaufnahmevorstellungen wurden 5133 (0,3 %) als *Suizidversuche*, 31.085 (2,1 %) als *psychiatrische Notfälle* und 34.230 (2,3 %) als Fälle mit einer *psychischen Symptomatik* identifiziert. 16,5 % der *psychiatrischen Notfälle* wurden so als *Suizidversuch *eingeschätzt. Unter den *Suizidversuchen* entfallen 53,4 % auf Männer und insgesamt 20,2 % auf die Altersgruppe der 25- bis 34-Jährigen. Alle 3 Syndromdefinitionen können über den gesamten Beobachtungszeitraum Fälle sowie deren zeitliche Variation abbilden.

**Fazit:**

Notaufnahmedaten zeigen Potenzial zur syndromischen Surveillance von *Suizidversuchen* und *psychiatrischen Notfällen* und bieten damit einen Ausgangspunkt für weitere Validierung und Analyse. Die Abbildung von Veränderungen in Echtzeit erweitert die bisherigen Forschungsmöglichkeiten zu psychiatrischen Notfällen in Deutschland. Eine systematische Surveillance von Suizidversuchen kann zu einer evidenzbasierten Suizidprävention beitragen.

## Abstract (en)

**Background:**

The occurrence of suicide attempts is a key indicator of the population’s mental health and therefore belongs in the domain of Mental Health Surveillance at the Robert Koch Institute. No data source is currently being used systematically for the continuous observation of psychiatric emergencies (including suicide attempts) in Germany. Therefore, the use of routine data from emergency departments will be explored in this work.

**Methods:**

We included routine data from 12 emergency departments between 1 January 2018 and 28 March 2021. We developed syndrome definitions for suicide attempts, psychiatric emergencies based on combinations of chief complaints, and diagnoses from patients presenting with psychopathological symptoms. A descriptive analysis over time was presented and stratified by age and sex.

**Results:**

In total 1,516,883 emergency department attendances were included, among which we identified 5,133 cases (0.3%) as suicide attempts, 31,085 (2.1%) as psychiatric emergencies, and 34,230 (2.3%) as cases with psychiatric symptoms. Among psychiatric emergencies, 16.5% presented because of a suicide attempt*. *Of cases presenting with a suicide attempt, 53.4% were male and 20.2% were aged between 25 and 34 years. Cases identified by all 3 syndrome definitions and their temporal variations could be displayed over the entire observation period.

**Conclusion:**

Syndromic surveillance using emergency department data indicates a potential for continuous surveillance of suicide attempts and psychiatric emergencies and provides a basis for further validation and analysis. The display of changes in real time extends the current research opportunities for psychiatric emergencies in Germany. Systematic surveillance of suicide attempts can contribute to evidence-based suicide prevention.

## Einleitung

Der Bedarf aktueller Daten zur psychischen Gesundheit der Bevölkerung ist in der COVID-19-Pandemie außerordentlich deutlich geworden [1, 2]. Aufgrund der großen Häufigkeit und Krankheitslast psychischer Störungen sowie der Potenziale der Förderung psychischer Gesundheit stellen diese Daten auch außerhalb von Krisen ein zentrales Feld der Gesundheitsberichterstattung dar. Da eine solche jedoch in vielen Ländern noch nicht systematisch aufgebaut ist, fordert der *Mental Health Action Plan* der Weltgesundheitsorganisation (WHO) zur Einrichtung nationaler Informationssysteme zur psychischen Gesundheit auf [3].

Im Jahr 2019 hat das Robert Koch-Institut (RKI) in Orientierung an internationalen Beispielen mit dem Aufbau einer „Mental Health Surveillance“ (MHS) für Deutschland begonnen [4]. Der Surveillance-Ansatz umfasst die kontinuierliche Erhebung, Analyse und Interpretation sowie Berichterstattung von Daten als Grundlage einer evidenzbasierten Planung und Evaluation von Public-Health-Maßnahmen zum Schutz und zur Förderung der Bevölkerungsgesundheit [5]. Für die Mental Health Surveillance in Deutschland trafen Expertinnen und Experten bzw. Stakeholder von Public Mental Health im Zuge eines systematischen Konsentierungsprozesses eine Auswahl von Indikatoren, die hoch relevante Aspekte der gesundheitlichen Lage abbilden [6], darunter neben vollendeten Suiziden auch Suizidversuche. Suizidversuche werden, im Vergleich zu anderen suizidalen Verhaltensweisen wie Suizidgedanken oder -plänen als stärkster Prädiktor vollendeter Suizide eingeschätzt [7, 8]. Daher kommt der Informationssammlung zur Häufigkeit und Verteilung von Suizidversuchen in der Bevölkerung eine zentrale Rolle in der Suizidprävention zu, wie auch deren Ernennung zu einem der Europäischen Gesundheitsindikatoren (European Core Health Indicators; [9]) belegt. Während Angaben zu vollendeten Suiziden aus der Todesursachenstatistik gewonnen werden können [10], steht zur Surveillance von Suizidversuchen bisher keine etablierte Datenquelle zur Verfügung.

### Erfassung und Häufigkeit von Suizidversuchen und psychiatrischen Notfällen

Um die Häufigkeit von Suizidversuchen in einer Bevölkerung zu schätzen, werden sowohl selbstberichtete Angaben von Suizidversuchen in repräsentativen Befragungsstudien erhoben als auch Routinedaten der Dokumentation medizinischer Behandlungen von Suizidversuchen herangezogen [8, 11]. Für Deutschland wird die Lebenszeitprävalenz von Suizidversuchen auf Basis eines repräsentativen Surveys (Erhebungszeitraum 2009–2012) auf 3,3 % geschätzt [12].

Wenn Teilnehmende in Befragungsstudien einen Suizidversuch berichten, kann dessen Angabe gegenüber anderen Datenquellen als vergleichsweise valide betrachtet werden. Allerdings ist die Durchführung von Befragungen insbesondere bei seltenen Ereignissen ressourcenintensiv und zur Erfassung kontinuierlicher Zeitreihen weniger geeignet. Zugleich ist von einer Unterschätzung der tatsächlichen Anzahl in der Bevölkerung auszugehen, da Personen einerseits nicht bereit sein könnten, einen Suizidversuch auch tatsächlich in der Befragung zu berichten (Reporting-Bias) und andererseits besonders hoch belastete Personen weniger bereit sind, an Studien teilzunehmen (Selection-Bias) [13].

Vorteile bieten demgegenüber Schätzungen der Anzahl von Suizidversuchen auf Basis der Routinedokumentation des Gesundheitssystems. Diese werden im Versorgungsalltag generiert und eignen sich bei strukturierter Erhebung für eine datensparsame und aufwandsschonende Mental Health Surveillance. Bei Verfügbarkeit der Daten in Echtzeit ist eine stetige und zeitnahe Detektion von zeitlichen Veränderungen unterschiedlicher Gesundheitsoutcomes möglich [14]. Routinedaten der medizinischen Versorgung, insbesondere von Notaufnahmen, werden bereits von einigen Ländern zur Surveillance von Suizidversuchen genutzt [11, 15] und ihre Nutzung wird von der WHO explizit empfohlen [16]. In Deutschland liegen aus Sekundärdatenanalysen einzelner Kliniken bereits erste Befunde für einzelne Berichtsjahre vor. Dabei wird die Gesamtzahl psychiatrischer Notfälle in Notaufnahmen pro Jahr auf ca. 1,5 Mio. geschätzt [17] sowie eine Prävalenz von 5–9 % angegeben [18–21].

Ein psychiatrischer Notfall wird definiert als ein medizinischer Zustand, bei dem das Vorliegen einer psychischen Störung zu einer gesundheitlichen Schädigung des Betroffenen und/oder einer Drittperson führt, sodass eine unmittelbare Diagnostik und Behandlung erforderlich ist [22]. Suizidale Verhaltensweisen werden zu deren häufigsten Ursachen gezählt [17, 18]. Entsprechend wird der Anteil von Suizidversuchen an allen psychiatrischen Notfällen in vorliegenden Studien mit 6–28 % angegeben [19, 21]. Während die Alters- und Geschlechterverteilung für Suizidversuche in Notaufnahmen bisher nicht beschrieben wurde, zeigt sich für psychiatrische Notfälle ein erhöhter Anteil an Männern (bis zu 62 %) und ein Durchschnittsalter von 40 bis 43 Jahren [18, 20, 21].

Insgesamt zeigt sich der Forschungsstand zu Suizidversuchen inkonsistent und lückenhaft sowie schwer über die Zeit vergleichbar aufgrund von Abweichungen in Falldefinitionen, Beobachtungszeiträumen und berichteten Kennwerten, welche z. T. ausschließlich in Bezug zur übergeordneten Gruppe der psychiatrischen Notfälle gemacht werden. Die Entwicklung einer systematischen und flächendeckenden Erfassung von Suizidversuchen bzw. psychiatrischen Notfällen auf Basis von Notaufnahmedaten wird daher explizit gefordert [17, 19]. Zu deren Entwicklung sollten Suizidversuche auf Basis von Notaufnahmedaten zunächst als Teilgruppe psychiatrischer Notfälle betrachtet werden, da so eine bessere Einordnung in die bisher vorliegende Literatur erfolgen und die Eignung der Datenquelle präziser eingeschätzt werden kann.

Seit 2018 pilotiert das RKI ein System zur Verarbeitung und Analyse von Routinedaten aus Notaufnahmen [23]. Zur Identifizierung spezifischer Surveillance-Indikatoren werden sogenannte Syndromdefinitionen genutzt. Als Syndrome werden die Sammlung von Symptomen oder klinischen Angaben und deren Zusammenführung in Kategorien bezeichnet [24]. Da Routinedaten nicht primär für Forschungszwecke erhoben werden, können mithilfe dieser Syndromdefinitionen jene Informationen aus der Notaufnahmedokumentation abgeleitet werden, welche für die Surveillance eines abgrenzbaren Anwendungsfalles benötigt werden [14]. Die Validität von Syndromdefinitionen determiniert als Basis der syndromischen Surveillance maßgeblich deren Nutzbarkeit bzw. Fähigkeit, Fälle mit hinreichender Wahrscheinlichkeit zu erkennen. Bisher wurden in diesem Rahmen Syndromdefinitionen für die Surveillance spezifischer übertragbarer Krankheiten wie gastrointestinaler Infektionen [25] und akuter Atemwegserkrankungen [26] entwickelt.

### Syndromdefinitionen für *psychiatrische Notfälle* und *Suizidversuche *in Notaufnahmedaten

Internationale Arbeiten fokussieren die Validierung von Syndromdefinitionen zur Abbildung diverser Anwendungsfälle psychischer Gesundheit, ohne jedoch den Prozess der Entwicklung detailliert darzustellen [24, 27, 28], sodass keinem standardisierten Vorgehen zur Erstellung von Syndromdefinitionen gefolgt werden kann. Zur Prüfung der Nutzbarkeit der am RKI verfügbaren Daten aus Notaufnahmen zum Zweck der Mental Health Surveillance von Suizidversuchen und psychiatrischen Notfällen ist es daher in einem ersten Schritt nötig, Syndromdefinitionen strukturiert zu entwickeln und dabei relevante Informationsquellen und vorhandene Codierungssysteme aus Deutschland einzubeziehen.

Ein Ziel der Arbeit ist die deskriptive Exploration und Auswertung von Syndromdefinitionen für die Surveillance von psychiatrischen Notfällen und Suizidversuchen. Durch den Vergleich mit der Literatur z. B. in Bezug auf die Häufigkeit von Fällen oder Fallcharakteristika soll weiterhin eine erste Einschätzung über die Aussagekraft der Syndromdefinitionen getroffen werden. Ihr Einsatz zum Zweck von Mental Health Surveillance wird in Bezug auf Stärken und Limitationen sowie weiteren Forschungsbedarf diskutiert.

## Methoden

### Setting und Studienpopulation

Für die vorliegende Arbeit wurden Daten aus der Routinedokumentation von Notaufnahmen genutzt, welche am ESEG-Projekt (Erkennung und Sicherung Epidemischer Gefahrenlagen; [29]) bzw. am AKTIN-Notaufnahmeregister [30] teilnehmen.

Als Einschlusskriterium wurde die Vollständigkeit der Daten mit lückenloser Erhebung für den gesamten Studienzeitraum berücksichtigt. Zusätzlich mussten in den Notaufnahmen entweder Diagnosen oder Vorstellungsgründe erhoben werden. Die Dokumentation in Notaufnahmen folgt in Deutschland keinem verpflichtenden Standard. Es sind mehrere Berufsgruppen (Pflege- und ärztliches Personal, Controlling) daran beteiligt und relevante Informationen liegen meist in unterschiedlichen Softwaresystemen vor [31]. Zur einrichtungsübergreifenden Auswertung wurden strukturiert vorliegende Daten aus ESEG und AKTIN zunächst in ein einheitliches, standardisiertes Format, entsprechend dem Notaufnahme-Kerndatenmodell (NoKeDa; [32]) überführt. Ein Datenpunkt im Datensatz entspricht einer Vorstellung in einer Notaufnahme. Wiederkehrende Notaufnahmevorstellungen von ein und derselben Person können nicht zugeordnet werden.

Folgende Variablen wurden berücksichtigt: Vorstellungsdatum (in Kalenderwochen und Monaten), Alter (in Altersgruppen), Geschlecht (männlich, weiblich), Dringlichkeit nach Manchester-Triage-System (MTS; [33]) oder dem Emergency Severity Index [34], Diagnose (International Statistical Classification of Diseases and Related Health Problems 10th Revision, ICD-10-Code als 4‑Steller; [35]) und Zusatzkennzeichen zur Diagnosesicherheit sowie Vorstellungsgrund nach MTS (besteht grundsätzlich aus der Variable MTS-Präsentation zur Eingrenzung des Beschwerdebildes und der Variable MTS-Indikator zur Spezifizierung des Symptoms) oder gemäß „Presenting Complaint List“ des Canadian Emergency Department Information System (CEDIS-PCL; [36, 37]). Während für die Dringlichkeit und den Vorstellungsgrund nach MTS und CEDIS-PCL jeweils nur ein Wert pro Notaufnahmevorstellung vergeben werden kann, ist bei der Diagnose die Vergabe von mehreren Werten erlaubt.

### Ethik und Datenschutz

Die im NoKeDa-Datenmodell vorgegebene Granularität der Daten ermöglicht eine anonymisierte Übermittlung an das RKI. Im Rahmen des ESEG-Projekts wurde ein positives Datenschutzvotum vom Datenschutzbeauftragten des RKI und vom Datenschutzbeauftragten des Landes Hessen eingeholt. Das Ethik-Komitee der Ärztekammer Hessen entschied, dass aufgrund der anonymisierten Natur der Daten kein Ethikvotum notwendig sei. Das AKTIN-Notaufnahmeregister erhielt ein positives Ethikvotum der Otto-von-Guericke-Universität Magdeburg (160/15).

Alle Notaufnahmen, die die Einschlusskriterien erfüllten, wurden um explizite Zustimmung zur Nutzung ihrer Daten für die Mental Health Surveillance gebeten. Die Nutzung der Daten aus dem AKTIN-Notaufnahmeregister wurde durch das wissenschaftliche Gremium genehmigt (Projekt-ID 2021-003). In diesem Rahmen wurde die in NoKeDa vorgesehene Granularität der Daten weiter vergröbert, um eine mögliche Reidentifizierung der Patientinnen und Patienten vollständig auszuschließen und deren besonderem Schutzbedarf Rechnung zu tragen.

### Syndromdefinition

Die vergebenen Diagnosen (inkl. Zusatzkennzeichen) und Vorstellungsgründe aus den Notaufnahmedaten wurden auf Werte durchsucht, die nach Einschätzung eines interdisziplinären Teams der Fächer Epidemiologie und Psychologie bei Hinweisen auf Suizidalität, auf einen psychiatrischen Notfall oder dem Vorliegen einer psychischen Symptomatik vergeben werden könnten. Zur Orientierung bei der Auswahl dienten ebenfalls deutsche Veröffentlichungen zur Erfassung von Suizidversuchen oder psychiatrischen Notfällen im Setting der Notaufnahme [18, 20, 21]. Zusätzlich wurden 5 eingeladene Notaufnahmeleiterinnen und -leiter zur Dokumentationspraxis bei psychiatrischen Notfällen und Suizidversuchen befragt.

Alle ausgewählten Werte wurden anschließend in Syndromdefinitionen zusammengeführt. Die Identifikation einer Notaufnahmevorstellung als Fall erfolgte, sofern mindestens einer der in Tab. 1 für die Variablen MTS-Präsentation/MTS-Indikator oder CEDIS-PCL oder ICD-10-Diagnose aufgelisteten Werte vorlag.

**Table 1:** 

Syndromdefinition Suizidversuche		Zusätzlich^a^ für die Syndromdefinition psychiatrische Notfälle		Zusätzlich^a^ für die Syndromdefinition psychische Symptomatik	
**MTS-Präsentation/MTS-Indikator**					
Selbstverletzung	Akute Atemnot, Gefährdeter Atemweg	Psychiatrische Erkrankung	Gefährdeter Atemweg, auffällige psychiatrische Anamnese	Auffälliges Verhalten	Auffällige psychiatrische Anamnese
	Auffällige psychiatrische Anamnese, auffällige Unruhe		Auffällige Unruhe, Schock, unzureichende Atmung		
	Auffälliger Verletzungsmechanismus, Schock		Hypoglykämie, mäßiges Risiko (künftiger) Eigengefährdung	Überdosierung und Vergiftung	Auffällige Unruhe
	Unzureichende Atmung, hohes Risiko (künftiger)		Mäßiges Risiko (künftiger) Fremdgefährdung, nicht		
	Eigengefährdung, stärkster Schmerz, mäßiges Risiko		Ansprechbares Kind, störend, veränderter	–	–
	(Künftiger) Eigengefährdung, nicht ansprechbares Kind		Bewusstseinszustand	–	–
	Mäßiger Schmerz, unpassende Vorgeschichte, unstillbare	Auffälliges Verhalten	Hohes Risiko (künftiger) Fremdgefährdung, mäßiges Risiko	–	–
	Große Blutung, unstillbare kleine Blutung, veränderter		(Künftiger) Fremdgefährdung	–	–
	Bewusstseinszustand	Überdosierung und Vergiftung	Auffällige psychiatrische Anamnese	–	–
Auffälliges Verhalten	Bericht über Überdosierung oder Vergiftung, hohes Risiko			–	–
	(Künftiger) Eigengefährdung, mäßiges Risiko (künftiger)	–	–	–	–
	Eigengefährdung	–	–	–	–
Psychische Erkrankung	Hohes Risiko (Künftiger) Eigengefährdung, mäßiges Risiko	–	–	–	–
	(Künftiger) Eigengefährdung	–	–	–	–
Überdosierung und Vergiftung	Hohes Risiko (künftiger) Eigengefährdung, mäßiges Risiko	–	–	–	–
	(Künftiger) Eigengefährdung	–	–	–	–
**CEDIS-PCL**					
351 – Depression/Suizidalität/absichtliche Selbstschädigung		352 – Angst/situationsbezogene Krise		354 – Schlafstörung	
752 – Einnahme einer Überdosierung		353 – Halluzinationen/Wahnvorstellungen		356 – Soziales Problem	
		355 – Gewalttätiges Verhalten/Fremdgefährdung		358 – Sonderbares Verhalten	
		751 – Substanzmissbrauch/Intoxikation		359 – Sorge um das Wohlergehen des Patienten	
		753 – Substanzentzug		360 – Kindliche Verhaltensauffälligkeit	
**ICD-10-Diagnose (G, V, Z, NA)**^**b**^					
X84.9 – Absichtliche Selbstschädigung		F00–F99 – Psychische und Verhaltensstörungen		T36–T50 – Vergiftungen durch Arzneimittel, Drogen und biologisch aktive Substanzen	
R45.8 – Symptome, die die Stimmung betreffen (inkl. Suizidalität & Suizidgedanken)					
				R44–R46 – Sonstige Symptome, die die Sinneswahrnehmungen und das Wahrnehmungsvermögen betreffen; Symptome, die die Stimmung betreffen; Symptome, die das äußere Erscheinungsbild und das Verhalten betreffen	
				Z03.2 – Beobachtung bei Verdacht auf psychische Krankheiten oder Verhaltensstörungen	
				Z73 – Schwierigkeiten bei der Lebensbewältigung	

^a^Die Syndromdefinitionen für „psychiatrische Notfälle“ und „psychische Symptomatik“ beinhalten auch alle Werte der jeweils links davon gelegenen Spalte(n)

^b^Zusatzkennzeichen zur Diagnosesicherheit: *G* Gesicherte Diagnose, *V* Verdacht auf, *Z* Zustand nach, *NA* Fehlende Angabe

### Deskriptive Auswertungen

Jene durch die Syndromdefinitionen erkannten Fälle wurden deskriptiv, stratifiziert nach Alter, Geschlecht und Dringlichkeit ausgewertet. Um die Fälle differenzierter zu charakterisieren und die interne Konsistenz einzuschätzen, wurden die 5 am häufigsten vergebenen Werte für die Variablen Diagnose und Vorstellungsgrund dargestellt.

Die Datenanalyse erfolgte mithilfe der Statistiksoftware R (Version 3.6.1) [38] und des Pakets *tidyverse* [39].

## Ergebnisse

Für den Zeitraum von 01.01.2018 bis 28.03.2021 wurde eine finale Studienpopulation von 1.516.883 Notaufnahmevorstellungen aus 12 Notaufnahmen inkludiert. Für alle erfassten Vorstellungen lagen jeweils Angaben zu Alter und Geschlecht vor. Informationen zur Dringlichkeit lagen für 89,3 % der Vorstellungen vor. 53,9 % der Fälle erhielten mindestens eine Diagnose, die Datenvollständigkeit für den Vorstellungsgrund lag bei 88,5 %.

Zur Identifikation von relevanten Fällen wurden 3 Syndromdefinitionen gebildet: *Suizidversuche, psychiatrische Notfälle* und *psychische Symptomatik* (Tab. 1). Die Syndromdefinition *psychische Symptomatik* beinhaltet alle Werte, die bereits zur Abbildung *psychiatrischer Notfälle* verwendet wurden, und jene, die als zu unspezifisch zur Abbildung *psychiatrischer Notfälle* galten. Dazu gehören bspw. die Diagnosecodes der Gruppen T36–50 und R44–R46, deren Einschluss aufgrund der Literatur erfolgte und durch die Befragung der Notaufnahmeleitenden bestätigt wurde. In der Gruppe der *psychiatrischen Notfälle* sind wiederum alle Werte der Syndromdefinition für *Suizidversuche* miteingeschlossen.

**Figure 1:**
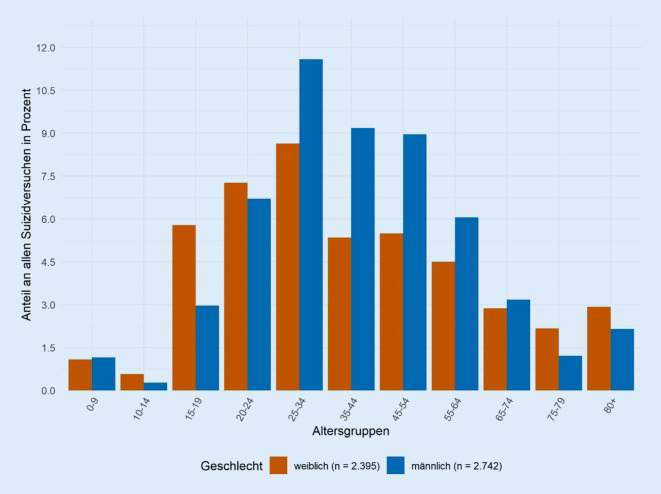

Unter Anwendung der Syndromdefinition *Suizidversuche* wurden 5133 Patientinnen und Patienten (0,3 % aller Notaufnahmevorstellungen) identifiziert (Tab. 2). 31.085 (2,1 %) Notaufnahmevorstellungen wurden als *psychiatrische Notfälle* klassifiziert und insgesamt 34.230 (2,3 %) Vorstellungen entsprachen den Kriterien der *psychischen Symptomatik*. Somit wurden 16,5 % der *psychiatrischen Notfälle* als *Suizidversuch* klassifiziert. Während 53,4 % aller Fälle von *Suizidversuchen* Männer betrafen, lag deren Anteil mit 58,9 % bei *psychiatrischen Notfällen* und 58,2 % bei *psychischer Symptomatik* höher. In der Gruppe der *Suizidversuche* wurden die meisten Fälle mit einer Dringlichkeitsstufe von 2 („sehr dringend“) codiert (24,8 %), während die Dringlichkeitsstufe 3 („dringend“) mit 25,9 % und 26,8 % bei den *psychiatrischen Notfällen* und der *psychischen Symptomatik* am häufigsten dokumentiert wurde. Insgesamt waren 45,9 % der Notaufnahmevorstellungen aufgrund eines *Suizidversuches* jünger als 35 Jahre und 48,9 % der *psychiatrischen Notfälle* wurden mit einem Alter zwischen 25 und 54 Jahren vorstellig (Tab. 2). Der relative Anteil weiblicher Fälle an *Suizidversuchen* ist im Jugendalter (15–19 Jahre) mit 5,8 % fast doppelt so hoch wie der Anteil männlicher Fälle (3,0 %) (Abb. 1).

**Table 2:** 

	Suizidversuche	Psychiatrische Notfälle	Psychische Symptomatik	Alle Notaufnahmevorstellungen
	*N* = 5133 (%)	*N* = 31.085 (%)	*N* = 34.230 (%)	*N* = 1.516.883 (%)
*Geschlecht*				
Weiblich	2392 (46,6)	12.761 (41,1)	14.292 (41,8)	733.514 (48,4)
Männlich	2741 (53,4)	18.324 (58,9)	19.938 (58,2)	783.369 (51,6)
*Alter*				
0–9	114 (2,2)	462 (1,5)	1151 (3,4)	173.377 (11,4)
10–14	43 (0,8)	480 (1,5)	550 (1,6)	51.487 (3,4)
15–19	449 (8,7)	2450 (7,9)	2614 (7,6)	61.674 (4,1)
20–24	717 (14,0)	3110 (10,0)	3332 (9,7)	88.206 (5,8)
25–34	1038 (20,2)	5236 (16,8)	5636 (16,5)	179.986 (11,9)
35–44	745 (14,5)	5009 (16,1)	5329 (15,6)	150.838 (9,9)
45–54	742 (14,5)	4987 (16,0)	5272 (15,4)	151.681 (10,0)
55–64	542 (10,6)	3826 (12,3)	4090 (11,9)	167.975 (11,1)
65–74	310 (6,0)	1935 (6,2)	2142 (6,3)	153.705 (10,1)
75–79	173 (3,4)	1137 (3,7)	1294 (3,8)	98.465 (6,5)
80+	260 (5,1)	2453 (7,9)	2820 (8,2)	239.489 (15,8)
*Dringlichkeit*				
1 – Sofort	53 (1,0)	311 (1,0)	336 (1,0)	19.497 (1,3)
2 – Sehr dringend	1271 (24,8)	7140 (23,0)	7490 (21,9)	155.283 (10,2)
3 – Dringend	1075 (20,9)	8050 (25,9)	9177 (26,8)	533.834 (35,2)
4 – Normal	1168 (22,8)	6844 (22,0)	7582 (22,2)	587.692 (38,7)
5 – Nicht dringend	236 (4,6)	1121 (3,6)	1288 (3,8)	58.003 (3,8)
Keine Angaben	1330 (25,9)	7619 (24,5)	8357 (24,4)	162.574 (10,7)

Die am häufigsten vergebene Diagnose innerhalb der Gruppe *Suizidversuche* war R45.8 („Sonstige Symptome, die die Stimmung betreffen – inkl. Suizidalität, Suizidgedanken“) mit einem Anteil von 12,9 % an allen vergebenen Diagnosen in dieser Gruppe (Tab. 3). Weitere 12,0 % der Diagnosen waren mit Alkohol assoziiert (F10.0 – „Akute Intoxikation“ und F10.2 – „Abhängigkeitssyndrom“). Für 66,0 % der Patientinnen und Patienten in der Gruppe *Suizidversuche* wurde der CEDIS-Vorstellungsgrund „Depression/Suizidalität/absichtliche Selbstschädigung“ vergeben, während in Notaufnahmen, die MTS nutzen, 64,0 % der *Suizidversuche* über die Variable „Selbstverletzung“ erfasst wurden. Am zweithäufigsten wurde entsprechend für 27,7 % der *Suizidversuche* „Einnahme einer Überdosierung“ (CEDIS-PCL) und für 24,7 % „Überdosierung und Vergiftung“ (MTS) als Vorstellungsgrund eingetragen. Die 3 häufigsten vergebenen MTS-Indikatoren für *Suizidversuche* waren „Unstillbare kleine Blutung“, „Hohes Risiko (künftiger) Eigengefährdung“ und „Mäßiges Risiko (künftiger) Eigengefährdung“.

**Table 3:** 

	*N*	%
**ICD-10-Diagnose**		
R45.8 (Symptome, die die Stimmung betreffen, inkl. Suizidalität und Suizidgedanken)	630	12,9
F10.0 (Akute Intoxikation – Alkohol)	396	8,1
F10.2 (Abhängigkeitssyndrom – Alkohol)	190	3,9
F33.2 (Rezidivierende depressive Störung)	170	3,5
F43.2 (Anpassungsstörungen)	170	3,5
**CEDIS-PCL**		
351 (Depression/Suizidalität/absichtliche Selbstschädigung)	2760	66,0
752 (Einnahme einer Überdosierung)	1160	27,7
751 (Substanzmissbrauch/Intoxikation)	121	2,9
352 (Angst/situationsbezogene Krise)	24	0,6
358 (Sonderbares Verhalten)	13	0,3
**MTS-Präsentation**		
Selbstverletzung	559	64,0
Überdosierung und Vergiftung	216	24,7
Auffälliges Verhalten	70	8,0
Unwohlsein bei Kindern	14	1,6
Besorgte Eltern	9	1,0
**MTS-Indikator**		
Unstillbare kleine Blutung	197	24,7
Hohes Risiko (künftiger) Eigengefährdung	193	24,2
Mäßiges Risiko (künftiger) Eigengefährdung	170	21,3
Auffällige psychiatrische Anamnese	90	11,3
Mäßiger Schmerz	55	6,9

^a^Da für die Diagnose mehrere Werte für eine Notaufnahmevorstellung möglich sind, beziehen sich diese Auswertungen nicht auf die Anzahl der Notaufnahmevorstellungen (*N* = 3653), sondern auf die Anzahl der codierten Diagnosen

Insgesamt wurden für *Suizidversuche* anteilig an den gesamten Notaufnahmevorstellungen pro Monat zwischen 0,2 % und 0,4 % Fälle erkannt. Für *psychiatrische Notfälle* und *psychische Symptomatik *bewegten sich Fälle anteilig zwischen 1,4 % und 2,2 % bzw. zwischen 1,5 % und 2,4 % (Ergebnisse nicht dargestellt).

## Diskussion

In der vorliegenden Arbeit wurden erstmalig Syndromdefinitionen für eine Mental Health Surveillance mit Notaufnahmedaten in Deutschland für die Indikatoren *psychiatrische Notfälle, Suizidversuche* und *psychische Symptomatik* erstellt und exploriert. Dabei konnte die prinzipielle Durchführbarkeit einer syndromischen Surveillance von *psychiatrischen Notfällen* und *Suizidversuchen* aufzeigt werden.

Der Anteil der *psychiatrischen Notfälle* an allen Notaufnahmevorstellungen fiel in der vorliegenden Arbeit mit 2,1 % geringer aus als in anderen Arbeiten, die diesen auf 5–9 % schätzten [18, 20, 21]. Der Anteil von *Suizidversuchen* an allen Notaufnahmevorstellungen lag in der vorliegenden Analyse bei 0,3 %. Während ein Bevölkerungssurvey der WHO aus dem Jahr 2013 eine 12-Monats-Prävalenz von 0,3 % berichtete [8], ermittelte eine Befragung in 74 deutschen Notaufnahmen einen Anteil der Suizidversuche von 2 % [19] und liegt damit über dem hier identifizierten Anteil. Wie hoch der Anteil von *Suizidversuchen* an der übergeordneten Gruppe *psychiatrischer Notfälle* ist, wird in anderen Arbeiten sowohl höher als auch geringer eingeschätzt. So wurden in der vorliegenden Analyse 16,5 % der *psychiatrischen Notfälle* als *Suizidversuche* identifiziert, während bei Kropp et al. [18] für 6 % und bei Freudenmann et al. [20] für 20 % (im Jahr 2000) bzw. 28 % (im Jahr 2010) der psychiatrischen Notfälle ein Suizidversuch als Vorstellungsgrund angegeben wurde.

Die Altersverteilung zeigte sich in Übereinstimmung mit der Literatur, in der ein Durchschnittsalter von 44 Jahren [18] und 39 Jahren [21] für *psychiatrische Notfälle* berichtet wurde. 20,9 % der *psychiatrischen Notfälle* waren unter 25 Jahren alt, was mit dem Befund von Kirchner et al. [21] zu ambulant verbliebenen psychiatrischen Notfällen übereinstimmt.

Eine Geschlechterverteilung von 58,9 % Männern und 41,1 % Frauen für *psychiatrische Notfälle* in der Notaufnahme wurde ermittelt, welche unterschiedlich zu den Verteilungen in den anderen Arbeiten mit 52 % Männern und 48 % Frauen [18] sowie 49 % Männer und 51 % Frauen [21] ausfielen.

In der Gruppe der *Suizidversuche* entfallen 12,0 % aller vergebenen Diagnosen auf alkoholbezogene Codierungen (F10.0 + F10.2), deren Relevanz im Setting der Notaufnahme in anderen Arbeiten ebenfalls aufgezeigt wurde [18].

Durch die Syndromdefinition der *psychischen Symptomatik* konnten nur 3145 (0,2 %) mehr Fälle gegenüber den *psychiatrischen Notfällen* identifiziert werden. Daher kann nicht von einer hinreichenden Trennschärfe dieser Differenzierung ausgegangen werden.

Zusammenfassend zeigen sich *Suizidversuche* als bedeutsamer und häufiger Vorstellungsgrund innerhalb der *psychiatrischen Notfälle* mit hier niedrigerer Anzahl identifizierter Fälle im Vergleich zu anderen Arbeiten. Die Verteilung *psychiatrischer Notfälle* über die Altersgruppen (gesamt und nach Geschlecht) stimmt weitgehend mit der Literatur überein. Abweichungen finden sich lediglich in den Geschlechteranteilen in Bezug auf alle Vorstellungen. Zudem konnte die Relevanz alkoholassoziierter Diagnosen für *Suizidversuche* im Setting der Notaufnahme repliziert werden. Bei der Interpretation und Einordnung gegenüber der Literatur ist zu beachten, dass Abweichungen auch durch den Einbezug der Daten mehrerer Kliniken in der vorliegenden Arbeit bei Vergleich mit Angaben aus meist nur einer Klinik in der Literatur sowie in der Auswahl eines anderen Beobachtungszeitraums begründet sein können.

Die Syndromdefinitionen können kontinuierlich Fälle abbilden und somit die Beschreibung von Veränderungen und Trends zum Zweck einer Surveillance ermöglichen. Dabei können Schwankungen der Fallzahlen im Zeitverlauf unterschiedlichste Ursachen haben, deren differenzierte Untersuchung über die Zielsetzung der vorliegenden Publikation hinausgeht. Besonders dringlich erscheinen in diesem Kontext die Analysen von Entwicklungen der Fallzahlen und Patientencharakteristika vor dem Hintergrund der COVID-19-Pandemie, die nachweislich zu starken Veränderungen im Versorgungsgeschehen der Notaufnahmen geführt hat [40, 41]. Ob und wie stark auch psychiatrische Notfälle davon betroffen sind, wird in bislang vorliegenden Arbeiten unterschiedlich eingeschätzt [42–45].

### Stärken und Limitationen

In Bezug auf die genutzte Datenquelle ist bei der Interpretation der Ergebnisse zu beachten, dass nur 12 Notaufnahmen eingeschlossen wurden. Deren Auswahl basierte auf freiwilliger Teilnahme [30] und ist somit ggf. nicht repräsentativ für alle Notaufnahmen in Deutschland. Zusätzlich ist das Notaufnahmekollektiv als Ganzes nicht repräsentativ für die deutsche Allgemeinbevölkerung (bspw. in Bezug auf die Altersstruktur und durch die Erfassung von Notaufnahmevorstellungen anstatt von Personen).

Bei der Beurteilung der Validität der Routinedaten in Hinblick auf die Abbildung des klinischen Geschehens ergeben sich außerdem folgende Limitationen: Die Dokumentation kann durch strukturelle Gegebenheiten in der Notaufnahme beeinflusst sein, was z. B. zu fehlenden Werten oder einer unvollständigen Datenübermittlung führen kann. Jegliche Änderung in der Dokumentationspraxis kann die Datenqualität beeinflussen und muss als Ursache veränderter Fallzahlen über die Zeit in Betracht gezogen werden [31]. Im Datenmodell NoKeDa ist zudem bisher nur die Nutzung von strukturierten Angaben vorgesehen, Informationen in Form von Freitextangaben (z. B. in der Anamnese) können nicht genutzt werden. Da Diagnosen und Vorstellungründe nicht für 100 % der Notaufnahmevorstellungen vorliegen, variiert die Wahrscheinlichkeit der Identifikation von Fällen je nach Vollständigkeit der beiden Variablen.

Zu einer möglichen Überschätzung von Suizidversuchen kann beitragen, dass schwere Selbstverletzungen (engl.: „self-harm“) als Suizidversuch gewertet wurden, auch wenn über die eingeschlossenen Codes ein suizidales Motiv nicht abgeleitet werden kann, da diese die Intentionalität nicht spezifizieren. Diese Schwierigkeit in der Datengrundlage ist bekannt [8] und wird z. B. im irischen Surveillance-System durch die Bezeichnung als Self-harm Registry reflektiert [46]. In Anlehnung an die dort getroffene Definition werden auch hier Handlungen von Selbstverletzungen mit variierend starker suizidaler Intention eingeschlossen. Nach Aussage einzelner Notaufnahmeleitenden werden Suizidversuche häufig mit der unspezifischen Diagnose R45.8 („Sonstige Symptome, die die Stimmung betreffen – inkl. Suizidalität, Suizidgedanken“) dokumentiert, wobei dieser Code Suizidalität nicht auf Suizidversuche eingrenzt. Möglicherweise erklärt dies die vergleichsweise hohe Anzahl von *Suizidversuchen* in der Altersgruppe 0–9 Jahre. Die Ergebnisse zur Dringlichkeit der hier identifizierten Fälle weisen allerdings darauf hin, dass es sich um gravierende Selbstverletzungen oder Suizidalität handelt. Zudem sind diese auch im Kindesalter nicht gänzlich auszuschließen [47, 48].

Weiterhin wurde im Rahmen des Austausches mit Klinikerinnen und Klinikern aus der Notaufnahme von einer eher zurückhaltenden Vergabe von Diagnosen psychischer und Verhaltensstörungen berichtet, da die Behandelnden mögliche negative Konsequenzen der dokumentierten „F-Diagnose“ (ICD-10 F00–F99 = Psychische und Verhaltensstörungen) für die Patientinnen und Patienten vermeiden wollten. Derartige Fehlklassifikationen müssen vor dem Hintergrund der Stigmatisierung und Diskriminierung von Menschen mit psychischen Störungen verstanden werden. Sie stellen eine allgemeine Schwierigkeit bei der Etablierung einer Mental Health Surveillance dar [49].

Da die Dokumentation in der Notaufnahme häufig auf die Erfassung der Verletzung und weniger auf die Ursache der Hauptbeschwerde fokussiert, wird grundsätzlich davon ausgegangen, dass psychische Störungen in der Notaufnahme nicht vollständig erfasst oder unterschätzt werden [50], zumal davon Betroffene vorwiegend nur dann vorstellig werden, wenn begleitende somatische Beschwerden auftreten. Auch für den Indikator der *Suizidversuche *ist anzunehmen, dass dessen Häufigkeit in der Bevölkerung höher liegt als im Setting Notaufnahme, da nur ein gewisser Teil der Suizidversuche zu Verletzungen führt, die medizinisch versorgt werden müssen. Auf der anderen Seite kann es vorkommen, dass ein Fall in der Notaufnahme noch als Suizidversuch gezählt wird, anschließend aber im stationären Setting verstirbt [8]. Zur Überschätzung der Fallzahlen kann außerdem beitragen, dass aufgrund der anonymen Datenstruktur in dieser Auswertung die Vorstellungen und nicht die Personen gezählt werden. Dabei ist bekannt, dass Personen mit psychischen und Verhaltensstörungen wiederholt vorstellig werden, insbesondere bei alkoholassoziierten Problemlagen [51]. Primäres Ziel der Notaufnahme-Surveillance ist demnach nicht die Identifikation von Indikatoren auf Einzelfallebene zur Darstellung von sektoren- oder bevölkerungsbezogenen Prävalenzen, sondern vordergründig die Beschreibung von zeitlichen Veränderungen in der Notaufnahme.

### Forschungsbedarf und Ausblick

Die Optimierung der Syndromdefinitionen durch weitere Validierungsstrategien am Beispiel internationaler Evaluierungsstudien [24, 27, 28] und unter Verwendung weiterer Datenquellen als Referenzwert (z. B. aus Rettungsdienst oder stationärer Behandlung) kann eine Unter- oder Überschätzung der identifizierten Fälle innerhalb der Notaufnahmepopulation verringern. Um die Vergleichbarkeit mit anderen Datenquellen zu verbessern, sollten die Analysen auf Kliniken beschränkt werden, die für alle Notaufnahmevorstellungen vollständige Daten liefern. Grundsätzlich könnten auch vertiefte Kenntnisse des Codierungsprozesses in Notaufnahmen zu einer Weiterentwicklung der Syndromdefinitionen (z. B. bzgl. des Einschlusses relevanter Variablen) beitragen. Der Einschluss weiterer Notaufnahmen in die Mental Health Surveillance kann die Repräsentativität der Daten stärken und eine umfassendere bzw. verlässlichere Abbildung der Indikatoren unterstützten.

Zum Zweck der Mental Health Surveillance erlauben die entwickelten Syndromdefinitionen die Abbildung von *Suizidversuchen* und *psychiatrischen Notfällen* in Echtzeit aus mehreren interdisziplinären Notaufnahmen in Deutschland. So können zeitliche Veränderungen in Echtzeit in mehreren Kliniken beobachtet werden. Diese systematische und kontinuierliche Erfassung von Indikationsbereichen der psychischen Gesundheit trägt insofern zu einer Erweiterung der derzeitigen Forschungsmöglichkeiten bei. Damit ist die Grundlage für den Auf- und Ausbau einer Surveillance gelegt und kann als Ausgangspunkt für vertiefende Untersuchungen von Trends dienen.

Sofern die Daten zur stetigen Beobachtung und differenzierten Analyse zeitlicher Veränderungen von *Suizidversuchen* genutzt werden, können Präventions- bzw. Interventionsbedarfe in spezifischen Personengruppen identifiziert oder auch mögliche Effekte von Maßnahmen abgebildet werden. Auf dieser Basis könnte z. B. ein Beitrag zur Evidenzbasierung des nationalen Suizidpräventionsprogramms [52] geleistet und damit im besten Fall langfristig die Krankheitslast durch Suizidversuche sowie Sterblichkeit an Suiziden verringert werden.
